# Methodologic Issues in Using Land Cover Data to Characterize Living Environments of Geocoded Addresses

**DOI:** 10.1289/ehp.0901863

**Published:** 2010-03

**Authors:** Paul A. Zandbergen

**Affiliations:** Department of Geography, University of New Mexico, Albuquerque, New Mexico, E-mail: zandberg@unm.edu

Estes et al. (2009) presented an interesting analysis of the relationship between blood pressure levels of individuals in four metropolitan regions and their living environments. Remotely sensed data was used to determine urban, suburban, and rural living environments as well as day/night land surface temperatures (LST). These remotely sensed data sets are readily available nationally, increasing the replicability and consistency of the methods.

Estes et al. (2009) characterized living environments using the 2001 National Land Cover Dataset (NLCD; Homer et al. 2004). Detailed land cover classes were reclassified into broad categories of urban, suburban, and rural, and the original 30-m resolution raster data was resampled to a 1-km grid using a majority filter to match the resolution of the LST data. Residential addresses were geocoded and their location compared to the 1-km grid cell values to establish the living environment variables. There are several problems that result from this particular methodology, which I address below.

First, Estes et al. (2009) geocoded the residential addresses using SAS/GIS geocoding software which employs TIGER data (SAS 2010) from the U.S. Census Bureau for street geocoding. The positional accuracy of TIGER data is not very good (e.g., Zandbergen 2008), and street geocoding in general is not very accurate (Cayo and Talbot 2003; Zandbergen 2009). The street geocoded location of the residence of a particular individual is therefore not very likely to fall inside the same 30-m grid cell as the true location of the residence. For example, the median error of typical street geocoding is in the order of 30–60 m for urban areas, about double that for suburban areas and much larger in rural areas (Cayo and Talbot 2003; Zandbergen 2009). This is likely to introduce a substantial number of misclassifications. Any point-in-raster overlay where the positional error of the points is of the same order of magnitude as the raster resolution is not very reliable, and the degree of misclassification will vary with the spatial heterogeneity of the land cover data.

Second, the positional errors in street geocoding are not random in nature. Typical street geocoding employs a standard offset from the roads in the placement of the geocoded locations. In many areas, however, the actual residence is located at much greater distances, especially in rural areas. In the 2001 NLCD land cover data, many rural and suburban roads are classified as developed open space. This means that geocoded rural addresses will typically fall on this land cover type, while the actual residence is located on an agricultural or vegetated category. This adds to the occurrences of misclassifications, especially between suburban and rural.

Third, the resampling of the original land cover data from 30 m to 1 km using a majority filter has the undesirable effect that small clusters of one land cover type that are surrounded by larger areas of other types will simply disappear. Estes et al. (2009) clearly acknowledged this and compared the classifications resulting from different resolutions; when resampling from 30 m to 1 km, only 63% of all locations were classified the same. This effect of resampling will vary between study areas. For example, urban development in Atlanta, Georgia, is relatively fragmented and the resampling results in a substantial reduction of the total area (from 2.0% of the study area in the original 30-m grid to 0.94% in the 1-km grid). A more compact urban development pattern such as Chicago, Illinois, is more robust to the effect of resampling.

The resampling does overcome some of the misclassifications introduced by the errors in street geocoding. In effect, the land cover type at the exact location of the geocoded address is no longer of greatest interest, and instead the “majority” land cover of the surrounding area is used. However, the effects of street geocoding errors and resampling will vary greatly between study areas, reducing the robustness of the final classifications of study subjects and introducing potential bias.

One approach to overcome some of these problems is to use the 2001 impervious cover data, which is provided as a complement to the 2001 NLCD land cover data. Imperviousness is classified between 0 and 100% and corresponds closely to the different land cover types, albeit providing more detail. The benefit of using impervious cover is that during resampling a simple averaging filter can be used instead of a majority filter. This type of filter produces unbiased results that are not dependent on the spatial heterogeneity of the landscape or the scale of resampling. Similar urban, suburban, and rural categories can be identified and will remain more robust under various resampling scenarios.

The availability of moderate to high resolution remotely sensed data at national and global scales is providing unprecedented opportunities to compare health observations to environmental variables, including land cover and climatic factors. When combining data from different sources, great care should be taken to ensure the accuracy of the input is sufficient to produce reliable results given the specific analysis methods employed. Street geocding in particular has been underestimated as a source of positional error. In addition, when resampling methods are employed to produce data sets of matching resolution, robust methods are needed to avoid the unnecessary introduction of noise and bias.
